# Contributions to the Physiology and Pathology of the Heart

**Published:** 1856-12

**Authors:** H. Bamberger

**Affiliations:** Prof. of Med. Clinic in Würzburg


### *Contributions to the Physiology and Pathology of the Heart.*

By H. Bamberger, Prof. of Med. Clinic in Würzburg.

(Translated for the Medical Examiner.)

### *On the Motion of the Heart.*

During the past summer, the rare opportunity was afforded me of closely observing through a wound in the parietes of the chest, the conditions of the heart's pulsation; a phenomenon frequently discussed, but as yet very obscure. The case occurred in a healthy man, 30 years of age, who attempted to take his life by stabbing himself in the breast with a sharp knife. The deed took place in a public garden, and I saw the patient about half an hour afterwards, when he was brought into the hospital. According to the testimony of those who brought him there, the bleeding had been profuse, and must at first have spirted in streams from the wound. He was pale and exhausted, but conscious. It was a smooth-edged, gaping wound, about an inch broad, inclining downwards, somewhat in front of the nipple, and at the lower side of the fifth left rib; upon each contraction of the heart, a considerable quantity of dark blood was discharged. It was evident that the patient, who belonged to the higher class, had intentionally selected that spot where the pulsations of the heart were best perceived. I pressed my index finger into the wound, and was greatly surprised to meet the flat, slippery point of the heart, which had, however, received no perceptible injury. There was scarcely a doubt that the pericardium was opened, as it would have been scarcely possible otherwise to have felt the point of the heart with the accuracy above described. Of course I availed myself of this favorable opportunity to study as far as was possible the motion of the apex of the heart. When my finger was introduced from the point towards the back, I could convince myself with the greatest certainty that at every systole the hardened and pointed apex of the heart slipped down along the front wall of the breast downwards, somewhat to the left, and a little below the lower margin of the wound; a copious discharge of blood taking place at the same time near my finger, whilst in the diastolic moment, the apex retreated upwards and could not be felt. The duration of the 1st period, when the point of the heart moved along my finger, appeared to be somewhat shorter than the 2nd period, yet I could make no positive assertion regarding this, as the contractions of the heart were so frequent, about 100 in a minute. Notwithstanding the strictest attention, I could not perceive the lever-like motion of the point of the heart, nor the rotation of the same about its longer axis. As regards the patient, it is merely necessary to state, that the suture was immediately applied. After a few days, pericarditis developed itself, with a loud, grating, friction sound, that lasted about ten days, accompanied by a moderate effusion into the left pleural sac. In spite of this condition, and of a slight hæmoptysis that occurred, the general symptoms were light; the patient rapidly recovered; the wound healed by the first intention, and after a few weeks he was discharged. Neither in the pericardium nor in the pleural sac, as daily investigations showed, did any admission of air take place.

It may be permitted me to offer a few remarks upon these observations. The most important object gained by it is, I consider, the establishment of the fact that during the systole of the heart, a true movement of its apex takes place in the direction from above downwards and towards the left. The question might arise as to whether this movement may not be considered only as an apparent one, induced by a systolic elongation of the heart; but since Harvey has shown more clearly the relations of the heart to the circulation, the previously accepted view of the heart's lengthening by the systole is entirely exploded, and at present the results of numerous vivisections and observations of Ectopia of the heart places beyond doubt the fact that the heart during the systole is lessened in its long diameter. The fact, therefore, that the apex of the heart can be felt considerably lower during its systole than during its diastole only by an actual depression of the whole heart can only be explained in the manner as described long since by Skoda. Skoda has published similar observations on a new born child, with deficiency of the sternum, where the fissure was only covered by skin. I had been a long time convinced of the correctness of Skoda's view, that in decided hypertrophy of the heart, the deeper position of the apex of the heart during the systole might be proved by percussion, and those observations further made it highly probable to me that similar relations existed for the normal condition; this probability has since become positive certainty. This circumstance explains also the fortunate results of the above mentioned case. If the communicated facts are considered, we are necessarily led to the view that the stab must have been made at the time of the diastole, for only on such a supposition is it conceivable that the apex of the heart, which was felt beating so distinctly in the wound, could remain uninjured. Besides it is not inconceivable that the violent physical concussion at the moment of stabbing may have prevented the occurrence of the systole.

How is it now with reference to the oft-mentioned lever-like motion of the heart, in consequence of which the heart beats against the parietes of the chest? Harvey, Cruveilhier, and Follin have observed this in Ectopia of the heart, and the numerous investigations of Volkmann seem further to place this fact beyond doubt. It may be imagined, that I am not inclined to oppose such authority, or to place too much value upon one negative observation, whilst at the same time I do not wish to undervalue its importance, as it appears to me to be the only one whose outward relations differ as slightly as possible from its normal condition. For it appears to me, that it can be readily conceded that the possibility of a lever motion of the heart may take place where the wall of the chestis absent or broken through, without its being necessary to maintain its actual occurrence in an uninjured thorax; it may be exactly as it is in the motions of the exposed brain, the possibility of which we can with justice deny in an uninjured skull; in one case as in the other a normal obstacle is absent, and forces are put in action which could not be so at an earlier period, although in each case they must have been present. So long as we are ignorant of the *quantum* and *quale* of the determining forces of the heart's motions, it will remain a useless task to determine *a priori* the direction of these motions; if we concede, however, the motion of the heart to be downwards, and consequently the existence of a force that drives it there, as is proved by the foregoing, then may we also grant the existence of another force, which has the tendency to move the heart lever-like forward. This, however, is so restricted by the chest-parietes, that the resulting motion is in the direction downwards, the heart moving downwards is pressed more strongly against the breast wall, which condition possibly assists the object of the heart's contraction. The lever motion can never of itself be the measure and the true reason of the heart's pulsation. For the greatness of these pulsations does not depend upon the material of the lever, upon its thickness, &c., but upon the length of its arm and the moving force. The heart's pulsation ought therefore, all other things being equal, be stronger in a giant than in a dwarf; in an adult than in a child; which all experience contradicts. On the contrary, it cannot be denied that the thickness of the heart's walls has a positive influence upon the force of the heart's pulsations, as daily experience in hypertrophy of the heart proves.

The complete parallelism that exists between the anterior surface of the heart and the interior wall of the chest, the perfectly flat surfaces of both, and the intimate contiguity of the same in the closed thorax, do not accord as Kiwisch has already shown, with the idea that the apex of the heart beats against the breast wall, and is forced into the intercostal spaces. In narrating later experiments upon animals which I have had the opportunity of making, I shall return again to the question of the lever motion. Though I cannot entirely agree with Kiwisch's view, and must hold to the motion of the heart in every case being downward and toward the left, yet I fully coincide with him when he makes the perceptible impulse of the heart depend not upon a peculiar beat of its apex against the breast, but merely upon the evident systolic hardening of the muscles of the heart pressed into the intercostal space. But it may be asked if this is the case, why is the pulsation of the heart felt only at a small spot corresponding to its apex, and not over the whole surface where the heart lies upon the breast-wall? Several things appear to me to contribute to this. First, that part of the heart, that is directly upon the breast wall, belongs entirely to the right ventricle, which on account of its comparative thinness, is far less fitted to make its systolic hardening outwardly apparent, whilst that point of the left ventricle that lies close upon the breast wall, from its greater muscular character is consequently better fitted to make its action apparent. Besides, we must not forget that the juxtaposition of the upper ribs which are closer even than the sternum, and more particularly the thick muscles of the breast, render it almost impossible to perceive the hearts's contractions under ordinary circumstances. I have myself often observed, in children and in emaciated persons, that the heart's impulses frequently occupy much greater space, and indeed can often be clearly felt wherever the heart touches. In hypertrophy of the heart, this is, as is well known, a daily phenomenon. I have seen very frequently the right ventricle of the heart giving as decided an impulse as the apex, and yet there exists no reason for supposing that the motions of hypertrophical hearts, leaving the strength out of the question, differ in any way from those of the normal ones. If the breast muscles of a rabbit be removed, and the intercostal space exposed, the pulsation will be distinctly felt on every part that the heart touches, though this could not have been previously perceived. There is, therefore, no necessity to postulate any other than the usual motions of the heart's pulsations.

Skoda (5te. Aufl. p. 162), opposes his view of the action of the contracting power of the lungs upon the chest parietes, to the views of Kiwisch. The conclusion of his argument purports as follows: "Since the heart is held in contact with the chest-parietes, and the diaphragm by the expanded lungs, and the contracting power of the lungs causes a continual contraction of the soft parts of the chest-wall, then the heart, whatever form it may take, can never by a change of form cause an arching of the intercostal space or diaphragm from above or below; it must rather, if there be no other influence upon its condition, produce a slight drawing inward of the intercostal space and of its diaphragm."

In spite of my great reverence for Skoda, I must here be permitted to differ from him. If we were treating merely of the diminished space of the heart during the systole, a contraction of the intercostal space rather than arching of the same would occur; but when on that account the heart passes into a more ball-like form, its muscular fibres hardening, and consequently producing a pressure against the chest-wall, which does not take place during the systole, then the question is, whether this pressure from without is sufficiently great, not only to prevent the contraction of the lungs, which acting through the heart produces a contraction of the intercostal space, but actually produces a positive rest. Already *a priori* this possibility must be admitted, since no other sufficient reason exists, and *a posteriori* shows even that the existence of a systolic arching is in fact the case.

On the rotary motion of the heart, mentioned by many experimenters, the consideration of this case offers no conclusions. Whilst it is at first sight probable that such a one can only be sufficiently clear on the bases of the heart, and not merely at its apex, and it were unjustifiable to deny the motion solely on the ground of one negative observation; on the other hand, the peculiar undulating arrangement of solid exudations in pericarditis confirms the same to a very great degree.

I have intentionally mentioned the results of my observations, and such as immediately grow out of them, without bringing into the question any experiments on animals, because I am of the opinion that any deduction from the phenomena of the heart's motions in animals can only be applied with great care to man. But I believe not the less, that they must yield exceedingly important data, if they are brought into harmony with the observations on healthy and diseased men. I have the liveliest desire to observe the phenomena of the heart's motion in animals, in a much more extensive manner than has yet been offered to me in man; but I also believe that an actual advantage can only be drawn from such observations, by preserving intact the relative position of the heart and lungs, because, from the beginning, I was convinced that any important disturbances in the normal relations must produce such considerable changes in the heart's motion that any application of the same could not be thought of.

Further observations have in the highest degree confirmed this view, and convinced me that in the open pleura, or in the removal or tearing out of the heart, its motions suffer the most important changes, and indeed not rarely are completely opposed to its normal condition. Too true it is, that the various investigations of extracted hearts have rather hindered than assisted the student on the motions of the same.—*Archiv für Pathologische, &c.*

